# Clinical Society of New York Polyclinic Medical School and Hospital

**Published:** 1912-06

**Authors:** 


### Clinical Society of New York Polyclinic Medical School and Hospital.

Meeting of April 1, 1912.

---

### Three Cases of Sterility Secondary to Adnexa Disease: Cured by Operation.

PRESENTED BY DR. HENRY V. HOLCOMB.

These three cases were of interest as showing what could be accomplished by conservative work.

The first case was a woman 19 years of age who had had a previous miscarriage in the sixth week of pregnancy, and gave symptoms showing pelvic trouble. Operation by Dr. Child showed both ovaries bound down by adhesions, a cyst attached to the left tube, and the tubes closed. The cyst was evacuated, the adhesions broken down, and the tubes probed with a fine bougie their entire length. Convalescence was uneventful. Four months after the operation she was free from any abdominal symptoms. Menstruation regular. She later showed symptoms of a floating right kidney, which was anchored by three silk stitches. Convalescence from this operation was also uneventful. February 2d, she had her last menstruation, and by the end of March she began to have morning vomiting with swelled breasts, and all the evidences of pregnancy. She had a precipitate delivery of a seven and a half months' fœtus, in the following August. The child died but the mother is alive and well.

The second case was somewhat similar. The patient came with all the abdominal symptoms of inflammation of the appendages. Dr. Child found on operation that both ovaries were prolapsed and bound down by adhesions, occluding the tubes. After her operation she returned home well. One month after the operation she became pregnant and was delivered of a full-time child.

The third case was a woman of 31 years of age who had been sterile for thirteen years. She had a former child and was anxious to have another. She had pelvic pains bilaterally, painful and profuse menstruation, which had been present for the previous four years. Examination showed a retroverted uterus, and tender appendages. Local treatment gave no relief. Operation in 1909 by dilatation, curettage, followed by laparotomy, showed the same adhesions of ovaries, with closed fimbriated extremities, as in the other two cases. The same method of treatment was followed, and the patient made an uneventful recovery. She gained in weight after the operation, and in the following November was delivered of a normal child.

None of the above three cases came to be operated upon for sterility, but Dr. Holcomb laid stress upon the desirable outcome of cases who have primarily adnexal trouble, and in the correction of this secured the desired pregnancy. Dr. Holcomb said that as far as could be ascertained these cases were free from gonorrhea.

Dr. Child said that the cases reported were very interesting to those who were striving for the correction of sterility where the fertility of the husband was unquestioned. He had felt that it was important to thoroughly probe the tubes in these cases to insure an absolute patency. With the adhesions of the adnexa corrected, malpositions replaced, and the toilet of the uterus complete, he thought that there was a field of work open to the careful and discriminating surgeon, which would be effective in overcoming some hopeless cases of sterility.

Dr. Holcomb, in closing the discussion, said that the tubes were absolutely closed and the fimbria clubbed so that in the ordinary course of events pregnancy would have been impossible.

### Specimen of an Aborted Right Kindey With Greatly Enlarged Opposing Kidney Non-functionating.

PRESENTED BY DR. CHETWOOD.

Dr. Chetwood illustrated the value of the colorimeter test in measuring the capacity of the kidney function. His patient on cystoscopic examination showed apparently normal ureteral orifices from both kidneys. The left ureter was easily catheterized, withdrawing urine of low specific gravity, deficient in urea and a number of leucocytes; pus though present was in insignificant amount. The right ureter was rigid and impossible to catheterize, and the cystoscope showed a certain amount of purulent granular material slowly exuding. The patient was then subjected to the colorimeter test, made by injecting 1 c.c. of phenol-sulphonephthalein, after having previously emptied the bladder. At one and two-hour intervals the urine was collected from the bladder. The returned percentage of color was so low that the individual kidneys were tested separately, resulting in an absence of color on the right side and a very low color return on the left.

Operative treatment seemed necessary, and after taking X-ray pictures to rule out the possibility of a calculus, the patient was operated upon. Operation showed a vestiginary right kidney with a ureter entering the bladder but having a sacculated distal end. On the left side which was unsuspected there was a perfectly normal ureter below, while above was a large ureteral sac almost as large as the small intestine, which entered the kidney. A large peri-nephritic pus sac occupied one side of the kidney, and practically no kidney tissue remained even in this organ to secrete. The patient could not possibly have lived as proved by the colorimeter test and subsequently verified by autopsy.

Dr. Sinclair said that in Dr. Chetwood's case there had been a slight previous injury many years ago, but aside from blood in the urine for a few days no recurrent symptoms of any kind had occurred.

Dr. Wyeth said a very interesting phase of the case was the extremely limited excretory area eliminating urea. Considering the good health the patient enjoyed, he thought the skin must have employed an important part in the excretion of urea.

